# The role of ‘familiarity’ and ‘normality’ in supporting transition to end of life care in paediatric oncology: A qualitative study

**DOI:** 10.1111/jan.16323

**Published:** 2024-07-13

**Authors:** Daniel Kelly, Mia Closs, Rachel McAndrew, Pam Smith

**Affiliations:** ^1^ School of Healthcare Sciences Cardiff University Cardiff UK; ^2^ Usher Institute University of Edinburgh Edinburgh UK; ^3^ Royal Hospital for Children and Young People NHS Lothian Edinburgh UK; ^4^ Department of Nursing Studies University of Edinburgh Edinburgh UK

**Keywords:** childhood cancer, emotions, end of life, family, supportive care, transition

## Abstract

**Aim:**

The aim of this study was to explore factors that helped when a child with cancer transitioned to end of life care in a hospital setting.

**Design:**

Qualitative exploratory design using reflexive thematic analysis.

**Methods:**

In‐depth, semi‐structured interviews were carried out with 7 sets of bereaved parents and 10 health professionals from one specialist paediatric oncology centre. Results were shared with professionals to help shape services in a new children's hospital.

**Results:**

Three themes were identified: ‘change and facing the unknown’, ‘the comfort of feeling normal’ and ‘knowing and being known’. Bereaved parents described a gradual awareness of the deterioration of their child's condition and the need for trust in health professionals. Professionals described the process as challenging but were guided by the needs of children and parents. Supportive and trusting relationships with professionals helped parents to cope with the transition.

**Conclusion:**

We identified practices that helped create a culture that supported parents and professionals involved in caring for children facing death from cancer. These were rooted in feeling supported and working to provide the best end of life care for children.

**Summary Statement:**

Given that the death of a child is a uniquely challenging event, this study indicates that the clinical setting can assist via the promotion of familiarity (supporting families over time) and normality (allowing family‐focused activities). These were helpful to parents and to professionals. However, professionals need emotional support when working with these families.

**Reporting Method:**

The study adhered to the Consolidated Criteria for Reporting Qualitative Research.

**Patient or Public Contribution:**

The project steering group included one bereaved parent (who was not involved in the study), one consultant paediatric oncologist and one hospital chaplain.

**Impact statement**

There is a lack of research that examines the features of clinical settings that can support parents and children when cancer cannot be cured. New data have been provided on the experiences of parents whose children are transitioning towards end of life care. The importance of a supportive clinical culture that promotes trust and normality, based on the needs of families, has been described here. These findings will be useful to oncology nurses and other stakeholders involved in supporting parents and families transitioning to end of life care for a child with cancer.

## 1 INTRODUCTION

Although many aspects of children's cancer care have improved greatly, others remain challenging; one of the most difficult being when active treatment fails or when a child's cancer recurs (Gage, 2012). Over recent years, mortality rates for cancers in children have decreased in Europe by 25%. However, this improvement has not been seen everywhere with one study indicating only a 2.8% decrease in childhood cancer mortality between 1990 and 2017 and based on WHO mortality databases. However, in Eastern Europe, where paediatric cancer care is still developing, there has been a 50% increase, with the highest figures in Romania (3.61 per 100,000) and Hungary (3.57 per 100,000) (Bertuccio et al., 2020). There are no gender differences apparent with child cancer deaths, and there are around 250 cancer deaths in children in the United Kingdom each year, where the present study was conducted. This equates to more than four deaths per week (Cancer Research UK, 2023). This figure supports the need to understand practices that assist parents, as well as exploring the role that professionals and care settings play and how they can help when a child is facing death from cancer.

Parental expectations and hopes for cure can make the transition from curative intent towards palliation and beyond highly emotional, and one that needs to be negotiated with tact and care. It is important to understand the process of transition that occurs in paediatric oncology contexts especially as competing time demands and multiple pressures on limited resources are now the norm (Friedrichsdorf et al., 2015).

Depending on the primary diagnosis, a child with cancer may deteriorate gradually and, as each treatment proves unsuccessful, the decision to discontinue active cancer therapy can be associated with feelings of failure and guilt for both parents and health professionals (Janiste et al., 2017). This is a uniquely challenging clinical situation in the paediatric oncology context that deserves to be better understood from an empirical perspective.

This paper reports on a qualitative interview study conducted in one specialist tertiary paediatric oncology service and explores how the transition from curative intent to preparing for the likely death of a child was experienced. The study aimed to describe the experiences of bereaved parents and health care professionals involved in this field of paediatric practice to better understand how the transition towards death from cancer can be made more bearable (Bruera, 2004).

## 2 BACKGROUND

In the Cancer Action Plan for Scotland (2016), the Scottish Government in its plan for Children's Cancer Services stated:

Care, where cure is not possible, is as important as every other aspect of treatment. (p. 7)

In 2023, this was updated in a new national plan (Cancer Action Plan for Scotland (2023‐2026), following the COVID‐19 pandemic, with palliative and end of life care being emphasized:

Ensure that supportive care, palliative care, care around death and bereavement are integral parts of the cancer journey that people and their families and carers experience. (p. 17)

Translating this policy into practice, however, poses unique challenges to childhood cancer providers who are presented with an increased array of complex treatments across specialist and local paediatric cancer services (Managed Service Network for Children and Young People with Cancer in Scotland (2016). Furthermore, families themselves are now more diverse meaning that blanket recommendations need to be avoided and implemented based on a family's unique needs and cultural expectations (Kelly & Kelly, 2013). However, an enduring assumption is that the family unit is static enough to function around the needs of the child (Borgstrom, et al, 2019). When life‐threatening illnesses, such as cancer, impact the family then support resources will be needed to assist members in coping with impending loss.

Seminal ethnographic studies, such as Bluebond‐Langner (1978), helped to reveal the subtle social factors that shape the culture of paediatric end of life care; and the insights that children can develop as they gradually learn of their likely fate from viewing the behaviours of those around them. Children themselves can learn from what is not said, as much as from what is, and should not be considered passive observers but as agents who play an active role in maintaining the emotional equilibrium of those caring for them.

This is a radical shift in thinking and underpins a uniquely provocative situation that challenges traditional models of palliative care (that are usually shaped on the needs of adult populations) but may not always be applicable to the needs of children with chronic or life‐shortening conditions such as cancer (Friedrichsdorf & Bruera, 2018). Responding to clinical deterioration is of relevance in paediatric oncology settings where a child's condition and prognosis can alter rapidly (Friedrichsdorf & Bruera, 2018; Gage, 2012). One particularly challenge issue is balancing the highly technical aspects of treatment in acute oncology settings, which can serve to reinforce curative goals, with the emotional response to the news of the impending death of a child and the need for effective and timely palliation (Kelly et al., 2000).

In terms of psychosocial support provision, a wide range of issues will influence how individual families deal with the circumstances encountered during the final phase of their child's cancer trajectory—including place of care, relationships with health care providers, the communication of bad news with those within their social circle (including other parents), and the availability of appropriate information (Ananth et al., 2015). Understanding how parents or other family members cope with the end of life phase of childhood cancer should provoke health professionals to question what helps in this situation (Kassam et al., 2015). Previous studies have reviewed parents' treatment decisions in relation to a child's fluctuating needs; and some have suggested the need to help professionals to support parents during this emotionally demanding process (Gao et al., 2016). Choice and control are two key concepts that have been found to be essential to enhance parents' coping strategies (Vangelisti, 2009). Importantly, emotional distress may occur when parents feel that their initial, and sometimes very early fears of their child's death are unacknowledged, or when their concerns are not listened to (Jaaniste et al., 2017). In this instance parents may look more to each other for support; and may rely on unofficial channels such as online fora to discuss possible treatment options, or to vent their emotions about a child's condition and their own unmet needs (Lewandowska, 2022) While understandable this may leave health professionals unaware and uninvolved (and sometimes feeling criticized either overtly or covertly), thus increasing their own feelings of anxiety about how best to deal with the situation when they engage with parents (Barak et al., 2008).

Alongside studies carried out in hospital settings, there are also expectations for paediatric end of life care to be provided at home. However, this must be balanced with the needs of a critically ill child returning home towards the end of life, and the impact of discontinuing cancer treatment as professionals may be considered to have ‘given up’ on them (Snaman et al., 2017). This is likely to be especially problematic when the child has been cared for in the hospital setting for long periods of time; a place which will have strong associations with the underlying hope of cure since the diagnosis was first made (Mack et al., 2006). Health professionals, therefore, need to assess the provision of adequate and appropriate support for the family unit before end of life care at home can be considered (Lovgren et al., 2016). However, such a decision may also be shaped by other factors such as the availability of appropriate resources (e.g. availability of respite for parents or hospice care at home). Professionals have remarked on the psychological advantages of end of life care at home; although concerns about family wellbeing are also important and reinforce the need for adequate preparation and support to be made available (Winger et al., 2020).

To translate available research into practice in end‐of‐life care for children there is a need to bridge research insights and the practical implications. Liben et al. (2008) recommended:

Individuals working in this field need to: clearly define the population served; better understand the needs of children with life‐threatening conditions and their families; develop an approach that will be appropriate across different communities; provide care that responds adequately to suffering; advance strategies that support caregivers and health‐care providers; and promote needed change by cultivating educational programmes. (p. 852)

Although Liben and colleagues made these recommendations some years ago, they remain relevant. To achieve this position, however, it is necessary to appreciate the subtle social processes, such as the relational aspects of care in childhood cancer contexts. Also included are questions around the nature of everyday supportive practices and whether the roles played by the professionals involved are helpful to these families.

## 3 THE STUDY

The study was designed to gain insights into the ways that bereaved parents and health professionals describe the transition to end of life care for children with cancer, and to identity factors that may be helpful (Bruera, 2004).

### 3.1 Aims and objectives

The focus of the study was the transition from active cancer treatment to end of life care for children with cancer. Whilst it was the child who was receiving cancer treatment it was the experiences of parents and health professionals, as they work together attending to the child's needs at a final stage of the cancer trajectory, that was our focus. The researchers were interested especially in the emotional experiences of parents and professionals during the transition process (Mack et al., 2006).

The study also aimed to highlight both effective and less effective practices in the transition from curative to end of life care and to seek recommendations to help inform supportive care for families, health care professionals and paediatric cancer services to enhance the care of children dying from cancer.

## 4 METHODS

### 4.1 Design

This was a qualitative in‐depth study involving two groups of participants: parents of children who had died from cancer within a timeframe of 1–10 years, and a group of health care professionals working in a specialist paediatric oncology unit in Scotland where the children of the parent group had received cancer treatment. A Steering Group oversaw the study and met on three occasions. Members included one bereaved parent (who was not involved in the study), one consultant paediatric oncologist, one hospital chaplain and the research team (DK, PS, RM and MC). The parent member helped us to scrutinize invitation letters, interview prompts and all other forms of communication sent to participants. This was to ensure that we had been sensitive to their situation and experience of loss.

### 4.2 Theoretical framework

The principles of reflexive theoretical thematic analysis (Braun & Clarke, 2006) were used for the investigation and interpretation of all transcripts to adopt an exploratory, rather than confirmatory, approach to the data. Based on the established transition theories developed by Van Gennep (1960), Meleis et al. (2000) and Kralik et al. (2006), particular interest was made of aspects of data that related to the following:

- The phases of transition (interpreted as Endings/Separation; Fallow time/Limbo; Re‐incorporation into a new life) in a trajectory from curative intent to palliative/end of life care.

The requirements for effective transition include awareness and acknowledgement of the nature of the change occurring, acquisition of information, social support systems, a strong connection with others and a heightened awareness of self. The process involved in health‐related transitions also includes appropriate awareness and acknowledgement, engagement with the change and its impact over time, as well as understanding the role of critical time points and external events (Kralik et al., 2006).

In our study, we characterized transition as a process of emotional adjustment that results from a significant change in clinical planning (to end of life care) in a protracted trajectory (of cancer treatment) that had previously been focused on a curative outcome.

#### 4.2.1 Study setting and recruitment

The study was conducted in a specialist paediatric oncology setting within a children's hospital in Scotland. Bereaved parents were approached using an initial contact letter; the wording of which was amended and negotiated following advice from the Research Ethics Committee. Professionals were recruited via an open invitation to all staff on the paediatric oncology unit using email, posters and word of mouth.

#### 4.2.2 Inclusion/exclusion criteria

Eligibility to participate in the study was defined by the following criteria for parents:

- Parent/s of children who had been treated for cancer with experience of the transition phase from curative to palliative/end of life care, that is, an active treatment phase had occurred prior to instigation of palliative/end of life care.
- A minimum of 1 year and a maximum of 10 years on from the death of their child.

The time limitation (between 1 and 10 years following the death of the child) was established to allow a reasonable amount of time to have elapsed before being approached, while still allowing for data to emerge that reflected current practice, as many professionals and services had been in place for over 10 years. Each of the parents was assessed for suitability in advance by the clinical team as described below. Exclusion criteria included an inability to speak English or if the health care team felt that they were unsuitable due to other circumstances, such as multiple bereavements.

No restrictions were placed on the professionals as long as they had experience of caring for children with cancer, either currently or in the past, who had not been cured.

#### 4.2.3 Participant selection and recruitment

Potential parent participants were identified from an existing database maintained by the hospital. The database was also screened for any parents who might not be suitable due to additional bereavements, or other relevant personal circumstances.

Participants were informed of the two stages of the study, although none were obliged to undertake both and were free to withdraw at any point.

Our intention was to recruit 10–20 bereaved parents. Thirty‐one parents were sent letters of invitation to their last known address. Seventy two per cent of these did not respond and no further contact was made. Seven parents contacted the research team; however, this took a prolonged period of time to achieve. Of the seven parents who expressed interest in participating, a total of 10 couples (seven mothers and three fathers) did take part after further discussion with the researcher (MC).

An information sheet was circulated to all professionals working on the paediatric oncology unit at the time inviting them to take part in the study. This invitation was open to all professionals and data collection was carried out separately from the parents.

#### 4.2.4 Data collection

Stage one involved in‐depth parent interviews with the researcher (MC) either alone or with another parent. Stage two was a follow‐up on the first interview using the same questions and clarifying issues of relevance to participants.

**FIGURE 1:**
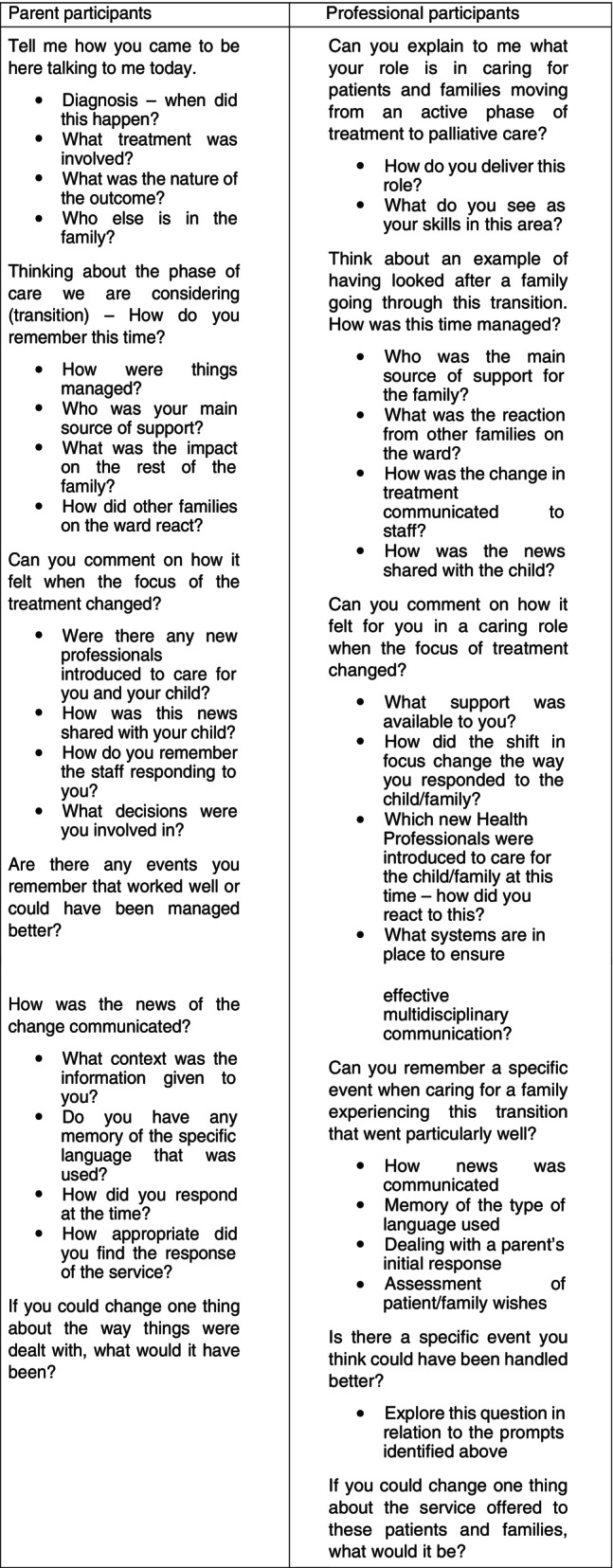
Questions and prompts used in the study for parents and professionals.

The researcher (MC) also conducted individual in‐depth interviews with each staff member. Interview prompts were used to focus the transition process with the professionals, and with the parents (See Figure 1). The emotional responses of parents in this study at the time of first being given news of their child's likely death has been reported on previously (Nelson et al., 2017).

Interview prompts were intended to ensure consistency across both samples and are included in Figure 1.

After the individual staff interviews had been completed, four professionals also volunteered to participate in a discussion group to explore preliminary themes emerging from the data. The sharing of these early results was intended to focus on the relevance to the study setting, rather than influencing the findings themselves.

Data collection took place over 24 months and totalled 18 h of parent interview audio recording. Just under 10 h, individual professional audio recordings were also achieved. Audio recordings were transcribed by a professional transcriber.

#### 4.2.5 Data analysis

Reflexive thematic analysis was employed. This was led by MC and the other research team members assisted in identifying key themes. An open coding procedure was used, with codes created and grouped as required to capture appropriately the experiences and issues raised during the transition of the child towards end of life care. Data from parents and health care professionals were analysed and reported separately, with insights from each group then combined when summarizing our findings. In this paper, we present data from both groups.

Interpretations from the data were also made over an additional series of meetings with the wider project advisory group. We continued to liaise with professionals to ensure that the findings were fed into service planning for a new children's hospital due to open in 2020.

#### 4.2.6 Ethical considerations

Ethical approval for the study was granted by the Research Ethics Committee of Edinburgh University (Ref. SS/0146) and with agreement from the local NHS Research and Development office. In reporting these findings, all children have been given pseudonyms to protect their privacy. No staff were named but professional groups are mentioned where relevant.

#### 4.2.7 Rigour and reflexivity

For purposes of rigour, all anonymized transcripts were made available to members of the research team with the data identified as representive of possible themes as well as an early draft of the corresponding section of the research report. Individual transcripts and findings were also shared with the professionals involved and with parents if they chose to see them. The research team worked in a highly reflexive way to support this process, and each other, during the study.

## 5 FINDINGS

### 5.1 Characteristics of participants

Of the seven bereaved families recruited to the study, six were married couples. One father in the married couple's group did not participate. Three fathers who had undertaken an initial interview declined further participation with only one father taking part in the second interview; reasons for refusal were not sought as the topic was highly sensitive. In the end, four parents (three mothers and one father) volunteered to take part in the second phase involving one further interview.

We achieved our aim to recruit 10 professionals, which included four doctors (two consultants and two associate specialists), five nurses (specialist practitioners, nurse managers and registered nurses) and one play specialist. Nine female and one male professional participated, which reflected the gender profile of the unit. Eligibility had been open to any professional who worked with children in this setting and had experience of supporting end of life care.

Parent participants reflected on the loss of seven children in total who had died following a cancer diagnosis and active courses of treatment. One child was almost 5 years old, one, a baby, having just turned 1 year and five were adolescents at the time of death. Five of the seven children were boys, and four had been an only child. Two of these were adolescents. The remaining two families, whose child had both been only‐children, the 5‐year old and the baby, had gone on to have other children subsequently.

The children who died had been diagnosed with a variety of childhood cancers including rhabdomyosarcoma, leukaemia and brain cancer, and all had died between 1 and 7 years after their initial diagnosis.

Professionals were invited to reflect on their experiences of caring for children transitioning from active cancer treatment to end of life care. Our findings are reported under three key themes:

1. Change and facing the unknown
2. The comfort of feeling normal
3. Knowing and being known

#### 5.1.1 Change and facing the unknown

##### The benefit of a sense of reality

The illness and treatment progression of many of the children whose parents participated in this study had occurred over some years and they talked about the benefit of having gained a level of familiarity with hospital routines, cancer treatments and medical terminology. Even in cases where the child had only survived 1 year from diagnosis through treatment to death, the treatment program had been so intense that the family had spent the first 6 months living in the hospital setting and soon became acquainted with the different oncology professionals as well as other families. Hospital routines and treatment protocols quickly became second nature. A father of one of the younger children commented:

After like three years of us being there more or less all the time we became quite used to the words they were using and everything just became, like, our life. Participant 2 Father

Becoming familiar with hospital routines, treatments and medical terminology appeared to provide parents with a sense of control and boosted their confidence in the professionals as well as enhancing their ability to cope.

Other parents emphasized how daunted they had felt at the time of the child's cancer diagnosis and how they had, with time and experience, gained confidence in their ability to predict, understand, and manage cancer treatments and associated complications. This was explained by one of the mothers:

… you got used to that because I thought, ‘How am I going to cope with this?’, and I was like, ‘Oh, this is just normal’, it's amazing how you really do cope with things … when they explain she'll have this in her, that in her, the next thing, and I'm thinking, ‘All these tubes, I'll never cope’, but you do, for some reason, well maybe it's just me, I don't know, but you do, you just cope you know, it's like, it doesn't end up being so scary or anything. Participant 2, Mother

However, the transition to end of life care presented a new and unfamiliar challenge and raised powerful emotions in parents, including fear and anxiety for themselves and for their child.

When asked what they thought might help most at this time one medical professional said:

I'd like to have more Palliative Care Nurses so we can offer time, time is so important. Families don't always want fancy drugs, they want your time, be it from me, be it from the nurses, it is about, you know, saying the sorry, saying, listening to them, more support, more time …

Whilst active treatment protocols had become familiar and predictable over time, the new shift towards planning for end of life care required parents to shift into a new role and focus, with unfamiliar challenges and responsibilities demanded of them—and of their child. Acknowledging this change, and the associated uncertainties, evoked powerful memories for the parents. One participant expressed this as a powerful sense of fear about what might be just around the next corner:

Scared for James, scared that he'd be in pain, scared that he'd be frightened, frightened's not there is it? … It's the same as scared I suppose … Yes, frightened for what he was going to be facing, and for what we were all going to be facing. Participant 3, Mother

Another parent also identified feeling scared when contemplating some of what her new role, as the mother of a child now facing end of life care, would involve:

I suppose scared for, I knew Georgia would be comfortable, I suppose scared about what were we going to have to face, what the future would be [interviewee becomes very emotional] scared I'd have to watch her die, watch her fade away in front of our eyes and not be able to do anything about it. Participant 2, Mother

The mother of an older boy described similar thoughts and concerns as intensely anxiety‐provoking. Her child was an adolescent, and her thoughts went more towards how he would react to being told of his poor prognosis and how she, as a mother, might support him. She recalled:

I suppose you're anxious for, anxious for Chris because he's my son and he's a teenager and I think because he understands everything that's going on and what they're talking about, it's anxiety for him … he was only thirteen, so he was a teenager, so anxious for how Chris's going to cope with this. Participant 4, Mother

This theme of a new reality illustrated how the parents, who had normalised their child's cancer treatment previously, now emphasized the emotions of fear and uncertainty that were taking over. In such circumstances parents sought solace in the normality of hospital routines as well as interactions with people around them, a theme that is explored next.

#### 5.1.2 The comfort of feeling normal

Participants talked of how their child's deterioration, and now poor prognosis, was a ‘monumental event’. It was experienced as heralding traumatic changes to almost every aspect of the family's life. Their experiences at this time indicated that, in response to the many changes now being faced, they came to prize anything that offered an opportunity to feel ‘normal’ again. Finding normality could be achieved in two ways; either by accessing some aspects of their ‘normal life’ or by being with other people or in an environment where their experience was considered ‘normal’.

For most participants this new ‘normal life’, and the stability it offered, meant being able to return to routines and habits of day‐to‐day life from before their child became ill. Some of this was achieved by being back in the home environment. For example, the joy of just being home and doing apparently insignificant but enjoyable things, was expressed in the following terms:

You'd get home and there was an elation ‘Yahoo! we're home! Let's go and just sit on the settee!’, you know, let's just, some you know, you quickly realise that humdrum routine things are what you actually aspire to. Parent Participant, Mother

Receiving comfort from aspects of life that were in keeping with ‘normality’, rather than a life shaped by hospital routines, was a common and positive experience expressed by these parents. Many lived a distance from the hospital so being home again was highly valued. For others, although living closer, found that their child became too ill or unstable to permit them to stray far from the hospital. In these cases, access to home‐like normality was achieved in the nearby family accommodation facility provided by a childhood cancer charity Young Lives versus Cancer Home from Home. All participants in this study had used and had been supported by this facility. During the interviews the normality that it provided was highly appreciated, particularly when compared to any other form of accommodation available from the hospital. As one of the fathers stated:

A brilliant facility … vital resource to have … I don't know how they choose the staff but certainly the staff were brilliant in terms of being able to empathise without going over the top, if that makes sense … they could, you know, they could relate to what was going on but there was a sort of, ‘Yes we're here to help but this is our job as well and therefore if you want us we're here, but if not we'll just get on with what we're doing’, so, and it takes a special type of person to be able to do that… it feels like home… Participant 1, Father

This promotion of normality, achieved by getting away from the hospital environment in order to feel ‘at home’, was expressed as being crucial to achieve respite and space from the new challenges they were now facing, and allowing the parents to regain some emotional resilience. Another of the fathers explained this further:

If you can supply a feeling of normal to somebody when they're under that level of, it's like, I'd equate it with like the soldier who's been up for thirty‐six hours, if you allow him a ten‐minute power nap you would not believe how much recovery can be achieved and realised in that power nap. Well equally, if you can give somebody a sense of normal, like the accommodation, or sort of the support that charities give and stuff like that you know, just being able to be as close to normal as you could maybe … having the opportunity just to be normal. Because normal is what you crave … if you can get to be normal, that's the best you've felt for a long time, getting to be normal, it's a challenge getting there, it really is but if you can supply normal, you're giving somebody the strength to recover and face the next battle. Participant 2, Father

The parents' accounts of valuing normality and the support offered by the professionals resonate with Kralik et al. (2006) who identified how strong connections with others, as well as good social support systems, are fundamental aspects of successful transitions in health contexts. For many this includes family and close friends, however, the unusual circumstances and demands that managing childhood cancer evoked meant that all the parents in this study had found that their biggest source of support came instead from the doctors, nurses and other staff within the hospital environment, rather than friends or family. One of the mothers expressed this shift:

We'd left all our friends and family here you know; we'd just been cut off from them completely and, you know, the nursing staff and the doctors and the people in (the charity house) they became our family and friends because they were your everyday, they were the people that understood what you were going through. Participant 3, Mother

##### The ‘new normal’

All participants expressed how health professionals and other families had become part of their ‘new normal’ life. They were also the people who had shared day‐to‐day experiences over the years, and were themselves familiar with what the families were now facing:

They've got hearts you know; they care what they're doing you know, they're not just doing it for the money are they you know? They're doing it because they want to save lives, you know, they're good people, you don't meet many people like that in your life unfortunately you know, but we did, I mean they pulled out every stop that they thought possible in medicine to try and save him you know, and then they were all at his funeral, Participant 4, Mother

For this group of parents, the health professionals and other families understood what it meant to have a child who was now seriously ill. It had become normalized and did not need to be explained. In both the clinical and family space, they felt supported as much as possible:

Mum For us our biggest support was the nursing staff, nursing staff and doctors, the whole hospital, cleaners, anybody. It was good to talk to somebody you didn't have to explain what anything was do you know…

Dad Aye, they knew.

Mum See people think we spent three years in hospital with him lying in the bed really ill and stuff like that, but it wasn't like that at all.

Parents emphasised the hospital staff having ‘hearts’ who cared about what they did, rather than ‘just doing it for the money’. One father appreciated their ability to empathize ‘without going over the top’, achieved by letting parents know ‘we're here to help but this is our job as well’ (in other words it was what they were paid to do, even though parents saw this as secondary to their motivation to care). The family accommodation facility was again mentioned as being like a ‘home away from home’. The staff's emotionally supportive style, coupled with availability of a home‐like atmosphere, helped to create a feeling that it could become ‘normal’ to have a child who was so seriously ill, and even dying from cancer. Furthermore, parents found the support from health professionals and other staff helped to create this ‘new normal’ which was in stark contrast to the burden of having to cope with extended family members who had not shared their experience of negotiating their experience.

Although understood to be well meaning, the interest shown by their extended family members was often experienced as placing additional burdens on parents, rather than being supportive. So many aspects of their hospital life that had become normal to them, but required to be explained to family or friends who were completely unfamiliar (and often horrified) with such experiences. One set of parents explained how, in these circumstances, they chose to keep relatives at arm's length and so eased the emotional demands placed on them to constantly explain what was happening. As this couple explained:

Dad Aye, none of the, the family didn't really understand, the family still don't understand.

Mum They don't understand what leukaemia is, we get it all the time, it's just something that's never been, nobody in the family's had it, both families, nobody's had it and they try, like the families and that's trying…

Dad It was getting annoying more than anything else.

Mum Aye.

Dad It was, it was hard work trying to you know, have them, they were there to support us, but you felt as if you were…

Mum You're physically drained because it was…

Dad Trying to explain everything to them.

Mum Aye, it was easier for us just to say to them, listen, don't come up for infection reasons, and stuff like that.

Other parents had also found that the emotional burden of coping when their child's condition had shifted from a curative focus to end of life care was too great a transition for extended family members to comprehend. While the parents felt that they had no choice but to accept the transition and to incorporate and normalize the changes and challenges they now faced, the wider family or social circle did not share the same understanding and came to terms with the new reality at different speeds. This left parents feeling isolated. Talking about the absences of communication and support from their extended family, one mother reflected:

I think sometimes I felt, I mean Peter's family just seemed to be, I think they, maybe, found it difficult to deal with it and cope with it and talk to us about it, … but I think, I think looking back you can't expect everybody to think the same as you anyway, and I think they found it really difficult to cope with. Participant 4, mother

She continued to discuss this experience with her husband:

Mum You can't really talk to people out with, out with [the hospital], it's a different world … it's a different world … eh, do you not think so (father's name)?

Dad It's hard to understand if you've not got a child that's like ill or … or a child that's got leukaemia.

#### 5.1.3 Knowing and being known

Knowing and being known in what had become a familiar environment was part of the transition experience, and it helped parents prepare for and choose how to best to cope with their child's end of life care. This required them to decide where they and their child would be best supported as the death drew closer. Parents talked about how, when faced with their fear and uncertainty, as well as the changes that had already occurred and the loss and grief that was about to come, they often sought the security of remaining in the hospital ward. This option was often raised by the professionals with parents and, where appropriate, with the child, when the change in prognosis and transition to end of life care was explained. For many the choice to stay was immediately clear with the preference to remain in what were familiar surroundings, and to be supported by professionals that they had grown to know so well over the years, and who had also grown to know them and their child. Familiarity was foremost in their thoughts at this time as it offered a supportive structure around which end of life care could even be contemplated:

We were asked what we wanted to do, go to [the hospice], come home or stay in the hospital, we chose to stay in the hospital because that was, that was his life, he, that's all he knew for the last three years and we were comfortable, the nurses were comfortable with him and stuff. Participant 3, Mother

A nurse participant also commented on the strong bonds that can be formed from the outset of cancer treatment and continue until the end of life phase:

The parents always remember the first person that they met. I've worked in the area coming up for nine years and parents that you'll see over in clinic will still say, you were the first nurse that I met when I came on to the ward.

Indeed, the preference for remaining in familiar surroundings with trusted and familiar people could make other options for end of life care provision difficult to contemplate. This was noted especially in relation to hospice care. Even though the facilities and staff in the hospice were known to be excellent, moving to an unfamiliar environment at this time was felt to increase the burden of the transition rather than ease it:

To have gone to new staff and new faces, like I was saying about it being different when we were on another ward, would have just been so difficult, I don't think I would have been, that would have made it even harder, we wanted the people that we knew and they knew us, they knew Izzie, you know, aye, I think it would have been much more difficult if we'd moved somewhere else, or even been at home. Participant 2, Mother

##### Professional support and the familiar

The professionals were also aware of this time of difficult choices, knowing that the hospice might provide more appropriate facilities, and a better palliative environment than the acute care setting, but they also appreciated the tremendous comfort that a sense of familiarity provided:

I actually think quite a lot of our parents choose to be in the ward because they know the staff and it's quite difficult when you've had an awful lot of treatment in a place to suddenly find a whole different team, unless it's going to be for a long period of time. Staff Participant

Furthermore, the role that familiarity played for families was expressed by some professionals as adding to their reticence in suggesting hospice care in case the parents felt pressurized or rejected, even when they believed it may be a better option. As one medical professional commented:

I think, as well, the families as well are used to the ward staff, and they've known us for so long that then initially going and thinking about going somewhere else you don't want them to feel like you're pushing them out the door. But on the other hand, you know, [the hospice] has got fantastic facilities and it might be the right place for some of these patients.

Not all the families, however, chose to stay on the ward and preferred instead to return home. Nonetheless, as the child's condition deteriorated and death became imminent, both the parents and children themselves might choose to return to the ward finding the company of people they knew, in a familiar environment, a vital source of support. Explaining the events of the night that their teenage son had died, one set of parents described this situation:

Mum He didn't feel very well eh, and he had, I had to phone the doctor and just say, ‘Actually Harry wants to go back into hospital’ … when I look back now, I actually think he knew, and he wanted to be in hospital and … although I would have loved him at home, and he actually felt it was home from home anyway just because he'd been with the nurses…

Dad Been in so long anyway.

Mum …and knew everybody, and (nurse's name) was on, and (nurse's name) was on…

Dad It was like his other family wasn't it almost?

Mum Yeah, all those nurses that were on that night, he felt really comfortable with them, he could joke with them and laugh with them, and it was like home from home, they were such a good team there.

As the bonds that formed within the unit were so strong, the news of cancer recurrence in children was soon felt by all. This was described by one nurse about a recent family:

I think it brings about quite a mixture of emotions for families, they want to be there for support but then they don't. Parents have told me in the past that they don't want to take themselves fully there because they don't want to take their mind to that point because that's almost imagining that they're in that situation, and I think sometimes they do toil with it.

##### Adrift in the unfamiliar

All the examples and experiences discussed in these data have illustrated the benefits from normality and familiarity for these families. In this sub theme, ‘adrift in the unfamiliar’, the experience of one mother further exemplifies the importance of the familiar at this time and the detrimental impact of ‘losing it’ through her experience of having her child transferred to adult services, at the same time of transition and her child's deterioration to a terminal condition. Reflecting on her experience, and trying to find the right environment and support for her and her son at the end of his life, she explained:

Maybe things wouldn't have been so hard for me in the end if I had had the support of the nurses and the doctors at [the paediatric hospital] at that point. We'd been battered from pillar to post from [their local hospital] who didn't have a clue about cancer … You know what I mean? … I pulled out every stop, sitting thinking on my own for hours at home, how to make it better for him, how to keep him normal you know, how not to be depressed, … but I really hit the floor … I had it double bad you know. And I couldn't pull myself, I couldn't, I didn't have the energy left to pull myself up out of that you know. Because of that last bit of the story, it was hard you know, that … just being cut adrift, that didn't help you know, I mind [remember] crying on the phone to [the consultant] and [he] is running about trying to do what he can to get Ben back and it didn't happen. Participant 3, Mother

While this was a single case and portrayed events that were different to the majority of parent or professional accounts, the negative impact of these events were significant and further highlight the importance that normality and familiarity played in end of life care for children.

Professionals also spoke of the importance of familiarity in forging strong bonds formed with families and how these meant that they remembered the children they had cared for:

Four of us were away at a meeting in Manchester the end of January and we got on the train to come home and of course it was quite soon after this family I'm going to see on Thursday, their little girl died. And we started a conversation, we spent the next four hours saying, ‘Do you remember X, Y, Z?’ Over the last ten years, we discussed every child that had died, and we were all in tears by the end of it and we thought do you know what, actually this has been one of the most therapeutic train journeys we've ever had. But I wondered if we could even say to the families that we still remember all your children fondly, and we had a story for every one of them. So, I would like to think that the transition goes okay every time…

## 6 DISCUSSION

These data provide new insights drawing into the experiences of our participants during the process of transition and the losses being faced; the roles that they now had to adopt in response to a child's worsening prognosis (Hinds et al., 2010). The first theme, *Change and facing the unknown*, revealed parents' experiences as they became aware of the transition to their new life situation marked with unfamiliar and uncertain challenges, new responsibilities and requirements demanded of them, and of their child. What was clear was the importance of subtle social practices (such as being called last in a clinic or using a ‘bad news room’) that were recognized by parents as harbingers of a poor prognosis (Nelson et al., 2017). Acknowledgement of this change in prognosis was the turning point for these parents, and they had acute awareness and expressed vivid physical memories, of this life changing event (Nelson et al., 2017).

*The comfort of feeling normal* illustrated ways in which parents strove for a sense of familiarity and comfort at such a challenging time; including spending time at home, deriving enjoyment from routine activities (such as simply sitting together as a family on the sofa) or being able to encounter home‐like experiences through the accommodation that was provided for parents by charities. The benefits of these facilities have also been evidenced in other research (Dyekjaer & Dreyer, 2019). All emphasized the positive impact that brief episodes of normality and strengthening of kinship bonds afforded them.

*Knowing and being known* demonstrated the importance the attachment to familiar surroundings and the support gained from relationships with health professionals that they had come to know and trust over the years, and who had grown to know them and their child. This support derived from a mutual respect and the trusting relationships that had built up over time. *Adrift in the unfamiliar*, presented a somewhat exceptional case example within the study that, nonetheless, further emphasised the importance of feeling supported by trusted people, in familiar surroundings and also the significance that this holds for parents whose child is dying of cancer. Experiencing a different clinical setting further exemplified how normality and familiarity acted as co‐existing supportive features of the culture that characterized this clinical setting.

The sense of familiarity with highly trusted professionals coalesced to create an environment that became supportive in its own right. While this may never have been verbalized overtly, it derived from the emotional and physical labour carried out by parents and professionals in this setting (Funk et al., 2017). Research conducted in an adolescent cancer unit, using ethnographic methods, also revealed the unspoken emotional support derived from a shared experience in a space that those who took part in helped to co‐create (Kelly et al., 2004). This was termed a ‘therapeutic milieu’ and these findings are echoed in the present study.

When considering our data there is a need to ask how best to recognize the lessons to be drawn. One way is to theorize the way everyone promoted a culture underpinned by a sense of familiarity and normality to support parents and children. Theoretical comparisons are also possible. For example, there is synergy with the suggestion of a ‘supportive habitus’ being created as defined by Bourdieu (1990, p. 53):

Objectively ‘regulated’ and ‘regular’ without being in any way the product of obedience to rules, they can be collectively orchestrated without being the product of the organizing action of a conductor.

In this explanation the individual agents (professionals, parents and even the children themselves) become active in the creation of this social world that can help support the imminent death of a child from cancer.

There are echoes of this collective action within our findings, and these data suggest that parents and professionals, who must deal with such an emotionally charged situation, cannot be considered passive agents in the face of enormous loss but instead choose how to orchestrate their emotions and behaviours to make them more bearable. They are actively involved in managing a situation that could be at risk of becoming emotionally out of control. To do so requires great skill on the part of the health professionals who support parents and children, while also supporting each other. They cope by carefully navigating though the powerful emotions of anticipated loss, and offer a calm and familiar sense of support to parents facing the death of a precious child.

These skills are particularly evident in the findings presented in the second theme: *The comfort of feeling normal*. Parents recognized that professionals had ‘hearts’ and cared about the work they did, rather than ‘just doing it for the money’. This has strong echoes with the work of Arlie Hochschild in her theory of how emotional demands are managed in the workplace, described in her seminal text ‘The Managed Heart’ (1983).

One father spoke of his appreciation of professionals' ability to empathize ‘without going over the top’, that is achieved by letting parents know ‘we're here to help but this is our job as well’ (in other words it was what they were being paid to do, even though parents saw this as secondary to their motivation to care). The accommodation facility for families, provided close to the hospital offered a valuable space as ‘a home away from home’. The staff's emotional response, coupled with availability of this home‐like facility, helped to create a feeling that there could still be some ‘normality’ in the highly unusual experience of having a child who is now dying from cancer. Furthermore, parents found that getting to know professionals well, often over a period of years, supported this ‘new normal’ which was in stark contrast to having to cope with extended family members who had not shared the experience of negotiating this new world.

Our data provide nuanced insights into the importance of ‘familiarity and normality’ as elements of the supportive culture found in this paediatric oncology setting. To this end, the study supports insights drawn from other research conducted in paediatric settings (Borgstrom et al., 2019) and in adolescent oncology (Pearce et al., 2020). The study also adds confirmation that the culture of clinical settings can be supportive (Kelly et al., 2000), but if this does not happen, they can also sometimes become brutalizing or even cruel (Layne et al., 2019). Within our data we also recognized elements of the transition theories mentioned earlier (such as anticipated Endings/Separation; experience of Fallow time/Limbo; and the incorporation of loss and bereavement in a new life).

### 6.1 Strengths and limitations

The study was carried out in one paediatric oncology setting with no opportunity for comparisons with other units. The sample was limited to those who agreed to participate and may have excluded those with other opinions, or different experiences. The sample was relatively small; but even this took time to achieve and may reflect the highly sensitive nature of the experiences being recalled. One strength was that the building of a new hospital meant that the findings were used to help shape support services for patients in the future. The COVID‐19 pandemic impacted by delaying the opening of the hospital but feedback was that our findings were found to be helpful when planning facilities for families. The dominance of women in the professional sample was unintentional but is noteworthy but may also be a limiting factor and could have introduced gendered bias. This was unintended as the invitation was open to all professionals. The lack of fathers within the parent sample is also noteworthy, as is the number who withdrew from the second interview. Again, this may reflect the sensitive nature of the project which took time and patience to conduct (Ananth et al., 2015).

### 6.2 Recommendations for future research

The nature of effective supportive and emotional care when cure is no longer possible, and the nature of the clinical settings in which end of life care for children takes place, are topics worthy of further enquiry. Importantly, insights gained from research such as this can also promote awareness of helpful and unhelpful clinical cultures, especially understanding the impact when emotional support to families is not available for a range of reasons including staff shortages or rising workloads.

### 6.3 Implications for practice, policy and research

The study confirms the need to better understand the features of the cultures that exist in hospitals that can, in themselves, support people facing suffering and loss. The impact of clinical cultures on other conditions and contexts should also be open to exploration. The therapeutic potential that can be found in promoting aspects of everyday family life for those in this situation is worthy of more attention. Health policy that promotes clinical environments that are focused on the needs of patients, families and professionals is needed. Finally, the provision of accommodation for families is only one example of how end of life care for children can promote normality as far as possible, even until the end.

## 7 CONCLUSION

This study has identified supportive features of clinical situations where children with cancer, and their families, are transitioning to end of life care. By recognizing these factors, it is possible to better understand the role that social and cultural practices in hospital contexts play in providing emotional support when the death of a child is likely. These factors include the value of ‘normality’ and ‘familiarity’ for parents who find themselves transitioning towards the end of life care for their child. However, professionals also require a supportive team culture to engage in such emotionally demanding work. Understanding the culture of care that is valued as the death of a child approaches may allow us to help when helping seems almost impossible.

## AUTHOR CONTRIBUTIONS

All authors have agreed on the final version and meet at least one of the following criteria (recommended by the ICMJE): (i) Substantial contributions to conception and design, acquisition of data or analysis and interpretation of data; (ii) Drafting the article or revising it critically for important intellectual content.

## FUNDING INFORMATION

This research was supported by The Edinburgh and Lothians' Health Foundation Fund, with additional financial assistance provided by Children's Cancer and Leukaemia Endowment Fund.

## CONFLICT OF INTEREST STATEMENT

The authors declare no conflicts of interest.

## Data Availability

The data that support the findings of this study are available on request from the corresponding author. The data are not publicly available due to privacy or ethical restrictions.
